# A Randomized Controlled Trial of Active Stretching of the Hamstrings and Core Control for Low Back Pain and Musculoskeletal Discomfort during Prolonged Sitting among Young People

**DOI:** 10.3390/jcm13175048

**Published:** 2024-08-26

**Authors:** Magdalena Plandowska, Marta Kinga Labecka, Aleksandra Truszczyńska-Baszak, Reza Rajabi, Maciej Płaszewski

**Affiliations:** 1 Faculty of Physical Education and Health in Biala Podlaska, Jozef Pilsudski University of Physical Education in Warsaw, 21-500 Biala Podlaska, Poland; magdalena.plandowska@awf.edu.pl; 2 Faculty of Rehabilitation, Jozef Pilsudski University of Physical Education, 00-968 Warszawa, Poland; marta.labecka@awf.edu.pl (M.K.L.); aleksandra.truszczynska@awf.edu.pl (A.T.-B.); 3 Department of Health and Sport Medicine, Faculty of Sport Sciences and Health, University of Tehran, Tehran 77871-31587, Iran; rrajabi@ut.ac.ir

**Keywords:** spine, pain, LBP, exercise, hamstrings, stretching, young adults

## Abstract

**Introduction:** Stretching exercises are important in both the prevention and treatment of nonspecific low back pain (NLBP). The aim of this trial was to determine whether an 8-week active hamstring stretching protocol combined with core stabilization and education regarding the maintenance of a neutral lumbar spine during activities could reduce NLBP and low back discomfort during prolonged sitting among young people. **Methods:** Participants (52 students aged 18–25) were randomly assigned to one of two groups: the Experimental group (the hamstring stretching group) and the Control group (only education). The intervention was conducted for 8 weeks. The primary outcome measures were pain intensity (VAS), low back discomfort (LBD), and functional disability (ODI). The secondary outcome measures were satisfaction with the intervention (GPE) and flexibility of the hamstring (SLR). **Results:** After the 8-week intervention in the E-group, results of VAS, ODI, and LBD were significantly lower comparing to baseline. In the C-group, no significant differences were observed. After the exercises program, there were differences between the E-group and C-group in VAS, LBD, GPE, and SLR tests (*p* < 0.05, *p* < 0.05, *p* < 0.05, and *p* < 0.0001, respectively). **Conclusions:** In conclusion, our study provides compelling evidence that an eight-week program of active hamstring stretching and core stabilization exercises can significantly reduce NLBP and musculoskeletal discomfort during prolonged sitting in young adults. These findings highlight the importance of targeted exercise interventions in managing and preventing NLBP, particularly among sedentary populations. Further research is warranted to confirm these findings and explore their applicability to broader populations and over extended periods.

## 1. Introduction

Decreased flexibility of muscles, poor posture, weak core stabilizing muscles, prolonged sitting in flexed positions, fatigue resulting from soft tissue overexertion, lumbar spine overload, and increased tone of spinal erector muscles might lead to an disturbance of lumbopelvic rhythm [1,2,3]. The pelvis is considered the base of the spine, and its anteroposterior position influences the sagittal curves of the spine [1,2]. Five muscle groups referred to as core stabilization muscles, including the erector spinae, hamstrings, gluteus maximus, abdominals, and hip flexors, support and maintain the pelvis in its natural position [4,5]. An imbalance of these muscles may cause the pelvis to tilt forward or backward [6]. Weakening of the core stabilization muscles leads to the activation of other stabilization mechanisms in areas distant from the spine, such as hyperactivity of the hamstrings [1,6,7].

Lumbar lordosis shape is influenced by the elasticity of the hamstring during hip flexion with extended knee [8,9,10]. The hamstring muscles are engaged in most daily and sporting activities, so maintaining their appropriate length is important. Shortening of hamstring muscles may have significant implications on the function and biomechanics of the knee and hip joints, as well as lumbopelvic rhythm [1,3]. One of the fundamental principles of lumbar spine pain protection is to maintain neutral spinal curvature while performing activities [11]. Limited range of motion of the hip joint and restricted flexibility in the lower extremities, particularly the hamstring, is associated with low back pain (LBP) [12,13].

The prevalence of nonspecific low back pain (NLBP) has increased significantly over the last twenty years, especially among young people [14,15,16,17,18], and the reasons for this include limited lower extremity muscle flexibility, weak core stabilization, and difficulties maintaining lumbar lordosis [19].

Even though there is no single solution to NLBP, stretching activities [5,20,21], and, more importantly, implementing an exercise routine that combines active hamstring stretching with hip flexion mobilization, as well as building the habit of proper hip flexion, which protect the lumbar spine might be beneficial in preventing and treating NLBP. Current LBP therapies frequently combine hip flexion techniques with general flexibility and strengthening exercises that are not focused on specific muscle groups, such as the hamstrings. On the other hand, it has been reported that school-based interventions are effective in promoting back health [22]. Consequently, implementing these interventions within school settings is recommended to address the high incidence of back pain among children and adolescents and to reduce the risk of adolescent LBP persisting into adulthood [23]. This underscores the pivotal role educational environments play in the early prevention and management of LBP, highlighting the need for targeted initiatives that enhance knowledge, posture habits, and physical exercise practices [22].

This study fills that gap by offering a focused intervention that may provide a more effective strategy to address NLBP and discomfort in young adults, a population increasingly affected by prolonged sitting time and sedentary behaviour, whether at work, during long classes, leisure time, particularly prolonged TV watching, computer/mobile use, and console-playing time. We decided to introduce a comprehensive but acceptable and feasible program for young people from so-called Generation Z, who have difficulties with long-lasting challenges. Hamstring stretching training could create an opportunity to practice techniques to maintain lumbar lordosis and the stability of the lumbar spine [3,11,20]. The aim of this trial was to determine whether an 8-week active hamstring stretching protocol combined with core stabilization and education regarding the maintenance of a neutral lumbar spine during activities could reduce LBP and low back discomfort during prolonged sitting among young people.

## 2. Methods

### 2.1. Study Design and Settings

This was a prospectively registered, single-blind, randomized, controlled trial. The Consolidated Standards of Reporting Trials (CONSORT 2010) recommendations [24] and the Standard Protocol Items: Recommendations for Interventional Trials (SPIRIT) guidelines [25] were followed when preparing the study protocol [26].

### 2.2. Participants

The participants of this study were students of physical education aged 18–25 from the University of Physical Education, Warsaw, Poland, in Biała Podlaska. We recruited them from the population of physical education students for convenience and for technical purposes, but also to minimize confounding factors, such as variations in the level and types of physical activity and other potential factors associated with low back pain, such as occupation, daily activities, and age.

Study eligibility criteria included NLBP that had been perceived for at least 3 months, no surgical history due to spinal problems, no previous experience of following a program of hamstring stretching exercises, and shortness of the hamstring muscle. We were interested in NLBP, in which it is not possible to identify a specific disease or structural reason to explain the pain [14]. Specific LBP (pain caused by a certain disease or structural problem in the spine or radiating from another part of the body due to underlying conditions such as spondylolisthesis, stenosis, malignancy, or inflammatory processes) [14], pregnancy, menstrual pain, leg length discrepancy over 1 cm, previous spinal surgery, presence of any contraindication to exercise, and medically verified chronic back disorders were the study exclusion criteria.

This study was conducted from November 2023 to January 2024 at the Faculty of Physical Education and Health, Jozef Pilsudski University of Physical Education in Warsaw, Biala Podlaska, Poland. The participants gave written informed consent before study procedures were initiated, in compliance with the Helsinki Declaration ethical guidelines [27]. The study was approved by the Józef Piłsudski University of Physical Education in Warsaw Senate Commission of Ethics (SKE 01-05/2023; 23 March 2023) and registered at Clinical.Trials.gov (NCT05995145, accessed on 16 August 2023).

### 2.3. Randomization, Allocation, and Blinding

Participants were randomly assigned to either the experimental group (hamstring flexibility exercises) or a control group (without any exercise intervention). A Microsoft Excel (Microsoft Office 365) random number generator was used for the randomization. The allocation was concealed using opaque, sealed envelopes, consecutively numbered and including each group’s name. We used the “RANDBETWEEN” Excel function for random number generation and then for generating the list of the participants set in a random order. Both the randomization and the allocation concealment were conducted by a researcher external to the study [25], and they were effective and not compromised. A single-blind trial (involving an assessor-physiotherapist with five years of clinical experience) was used. An evaluator who was blind to the group completed baseline and post-intervention measures, as well as measurement assessments. The statistician performing data analysis was blind to group allocation and interventions. Because of the nature of exercise programs, participants could not be blinded.

### 2.4. Interventions

The hamstring flexibility exercise program (Experimental group—E group) was conducted five times a week, 20 min per session, for 8 weeks. The intervention was divided into two parts: exercises with a massage foam roller followed by three active hamstring stretching exercises [26]. Participants performed hip flexion mobilization to simultaneously learn the correct habit of hip flexion, maintaining a neutral spinal position of the lower spine. The exercise program was based on the education of participants, individual exercises at home, and regular group meetings for follow-up physiotherapeutic control visits. The participants independently regulated the level of exercise difficulty based on their physical condition. The equipment used in the active hamstring flexibility program consisted of a foam massage roller and a gym mat. Throughout the intervention, everyone received the same type of foam massage roller.

During the first week of the intervention, participants were engaged in a rigorous four-day program that combines educational sessions with practical training focused on managing lower spine health. The program covers anatomy education, providing in-depth lessons on the structure of the lower spine, including vertebrae, discs, muscles, and nerves, with the aid of visual materials like diagrams and models to enhance understanding of spinal function and its role in movement and support. Postural instructions were also emphasized, offering comprehensive guidance on maintaining correct posture during various activities such as sitting, standing, and walking, along with ergonomic advice for optimizing workspace setups to promote spine health. Practical demonstrations illustrated proper techniques for lifting, pushing, and pulling to prevent back injuries, emphasizing effective use of leg and core muscles while maintaining spinal alignment. Participants received advice on integrating safe movement practices into daily tasks like bending, carrying objects, and performing household chores, facilitating the application of newly acquired knowledge in their everyday routines.

In the subsequent weeks, the focus shifted towards prescribed home exercises. To ensure that participants were performing the exercises correctly and consistently, the first check-up with the physiotherapist was scheduled two weeks after the intervention began. During this session, the physiotherapist reviewed the participants’ techniques and adherence to the exercise regimen. A second check-up was conducted four weeks after the start of the program, allowing the physiotherapist to re-evaluate and make necessary adjustments to the exercises.

Participants were required to register their exercise frequency in agenda sheet to track their progress. To further encourage adherence to the intervention, a combination of exercise logs, text messaging, and regular meetings were utilized to monitor and support the experimental group’s commitment to the program. This multi-faceted approach aimed to ensure that participants remained engaged and motivated throughout the intervention.

Participants in the control group (C-group) received an educational booklet and performed their regular baseline activities without a specific lower extremity flexibility program. Participants were contacted once a week by email and/or telephone. Participants were asked to continue with their regular routines and not receive physical therapy or other medical care.

The control group was given the option of participating in an exercise program with active hamstring stretching exercises combined with core stabilization and education regarding the maintenance of a neutral lumbar spine during activities after the final assessment was completed. This opportunity was taken into consideration by sixteen participants.

In a recently published study, protocol procedures in both groups have been described in detail [26].

### 2.5. Outcomes

Before random assignment, participants filled out the survey with questions about basic demographic data, their current physical activity, their medical history (including injuries), surgeries due to spinal ailments, and the frequency of NLBP within the last three months.

The primary outcomes were average pain intensity—Visual Analogue Scale (VAS) [28]; functional disability—revised Oswestry Low Back Pain Disability Index (ODI) [29]; perceived low back discomfort during prolonged sitting (LBD)—Borg CR-10 scale [30] assessed at baseline and immediately after the 8-week intervention; and global perceived improvement—Global Perceived Effect (GPE) scale [31], which was only evaluated after the 8-week intervention. The secondary outcome measure was the flexibility of the hamstrings (SLR test) [32], and this was also collected after the 8-week intervention. A detailed description was provided in the study protocol [26].

### 2.6. Sample Size

The required minimum sample size was 44. Thus, to manage potential drop-outs, 48 participants were enrolled. The sample size was obtained using the G*power program assuming a medium effect size of repetition and group interaction (d = 0.5) at a significance level of 0.05 and a statistical power of 0.85.

### 2.7. Procedure

Eligible candidates, who signed informed consent to participate, were enrolled to the study. A blind assessor-physiotherapist assessed the qualifying criteria. Before participants were randomly assigned to groups, they completed a baseline evaluation. An outcome assessment was conducted at baseline at the start of the study and immediately after the 8-week intervention.

A pre-screening questionnaire was administered before the start of the trial. The Formetric 4D rasterstereographic system (DIERS, International GmbH, Germany) was used to measure spinal curvature. The test was conducted in compliance with the protocol given by the device’s manufacturer [33]. Each participant underwent an individual examination. We analyzed kyphotic angle [°] (the angle between the C7 and Th12/L1) and the lordotic angle [°] (the angle between the surface tangents of the Th12/L1 and L5-S1).

The baseline examination included the following: VAS, ODI, LBD, and SLR test. The detailed procedure was described in the protocol [26].

### 2.8. Statistical Analysis

Statistical analyses were conducted using Statistica 14.0.0.15 (TIBCO Software Inc., Palo Alto, CA, USA, 2020). Before the data analysis, a normality test (Shapiro–Wilk test) was performed for all variables. Descriptive statistics of the variables were recorded (mean ± SD). The chi-square test was used for categorical variables. The Mann–Whitney U test was used to compare the groups. Each group’s descriptive statistics (Experimental vs. Control) were calculated independently. The baseline characteristics of the subjects were compared using the Student’s *t*-test. The Wilcoxon signed rank test was used to compare parameters (VAS, ODI, LBD, and SLR test) before and after the intervention. The E-group vs. the C-group difference (before and after the intervention) was compared using the Mann–Whitney U test. The delta symbol (Δ) was used to indicate the change in VAS, ODI, LBD, and the SLR test values after intervention. Positive delta values indicate a decrease in the values of the VAS, ODI, and LBD parameters and an increase in the values of SLR test. The a priori α significance level was set at *p* < 0.05.

## 3. Results

**Figure 1:**
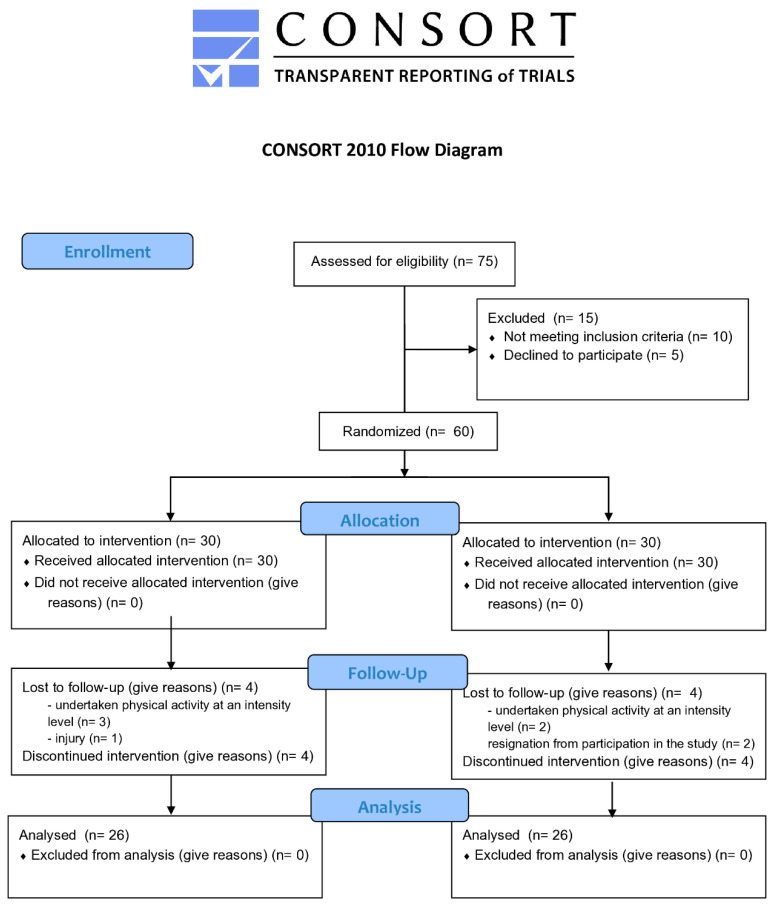
Flow diagram of participants.

Recruitment began in November 2023, and the post-intervention data were collected in January 2024. Out of 75 volunteers, 60 individuals met the inclusion criteria and agreed to participate in the study; 30 were allocated to the E-group and 30 to the C-group. Fifty-two (86.7%) completed the whole intervention. Eight were lost to follow-up because they discontinued the intervention and did not return for evaluation. The flow diagram of participants is shown in Figure 1.

### 3.1. Pre-Intervention Group Comparison

Table 1 presents the characteristics of the participants who completed the study and were included in the final analysis. No statistically significant differences between the groups (E- and C-group) were observed before the intervention.

Most participants from both groups reported experiencing pain very rarely (61.5% of the E-group and 46.2% of the C-group) or a few times a month (34.6%; 46.2%; respectively) (Table 2).

Analysis of the circumstances in which NLBP occurs showed that the three primary activities linked to NLBP by participants were sitting (69.2% of the E-group, 69.2% of the C-group), standing (61.5% and 46.2%), and lifting heavy objects (30.8% and 30.8%). Of the E- and C-groups, 7.7% and 23.1%, respectively, reported not knowing how to sit properly. In the E-group, 46.2% of participants reported knowing the rules and being able to apply them, and in the C-group, 46.2% said the same (Table 2).

### 3.2. Pre- and Post-Intervention Comparisons

Table 3 presents the pre- and post-intervention differences in the outcome measures. Significant decreases in VAS, ODI, and LBD were observed in the E-group (*p* = 0.0458, *p* = 0.0479, *p* = 0.0137; respectively). The SLR test results of this group also improved significantly (RL *p* = 0.000, LL *p* = 0.0000). There were no statistically significant differences between the pre- and post-intervention VAS, ODI, or LBD scores or SLR test results of the C-group.

### 3.3. Post-Intervention Results between Groups

Significant changes were seen in the post-intervention inter-group VAS, LBD, SLR test, and GPE. In the experimental group, values of VAS and LBD decreased, and the SLR test values increased significantly. There was no statistically significant difference in ODI (Table 4).

The global perceived improvement was dichotomized into “improved” (GPE scores 1–2) and “not improved” (GPE scores 3 to 7). A total of 61.5% of participants in the E-group confirmed the improvement after the intervention.

## 4. Discussion

The aim of this trial was to determine whether an 8-week active hamstring stretching protocol combined with core stabilization and education regarding the maintenance of a neutral lumbar spine during activities could reduce LBP and low back discomfort during prolonged sitting among young people.

Low back pain, especially nonspecific, chronic, primary low back pain, is a major health issue worldwide [14,15,17,18]. About 619 million people live with LBP. It is recognized as the most prevalent musculoskeletal condition, with serious public health consequences. As a primarily cause of disability worldwide, LBP not only may lead to pain, decreased functioning, and limitations on quality of life and societal participation but also impacts productivity on a global scale, thus placing a substantial economic burden on individuals and societies [14,15,17,18]. Importantly, it is a condition with great potential to be alleviated by rehabilitation interventions [16,18].

Addressing NLBP during the school stage is crucial, as early prevention and management can significantly reduce the risk of chronic pain and disability later in life [22]. Implementing preventative measures and educating students about proper posture, physical activity, and ergonomic practices can play a vital role in mitigating the onset of NLBP [22,23]. The importance of school-based interventions in this context cannot be overstated. Research has demonstrated their effectiveness in promoting back health, making them essential for reducing the high prevalence of back pain (BP) in children and adolescents and for mitigating the risk of adolescent NLBP persisting into adulthood [22]. Preventing NLBP at this stage is critical, as it is a condition with great potential to be alleviated by rehabilitation interventions [22,23]. School health programs that integrate strategies to improve knowledge, posture habits, and physical exercise not only enhance immediate health outcomes but also foster long-term musculoskeletal health and well-being that students can carry into adulthood [22,23].

Specific causes of primary NLBP include limited lower extremity muscle flexibility, weak core stabilization, and difficulties maintaining lumbar lordosis, especially during prolonged sitting [19,34,35,36]. Some studies have not found significant changes in pain and function after stretching interventions [34,37]. One of the reasons for these null results may be the fact that hamstring stretching without stabilization of the lumbar spine can increase the flexibility of the lumbar erector spine rather than the hamstring [38]. Consequently, stretching in this manner could cause excessive lumbar motion and increase lumbar flexion during forward bending tasks, and it may therefore increase the risk of injury to the spine from mechanical stress, eventually resulting in recurrent episodes of NLBP [39,40]. Pelvic position can influence hamstring stretching because the hamstring has its origin at the ischial tuberosity of the pelvis. The performance of lumbopelvic stabilization directs the stretching force to the hamstring, resulting in increased flexibility and preventing the lengthening of the lumbar erector spinae. In other words, specific hamstring stretching avoids excessive motion of the lumbar spine [38,41]. The relationship between lumbar lordosis and the efficacy of erector muscles was confirmed in the laboratory ultrasound imaging study by McGill et al. [11], especially the ineffectiveness of trunk stabilator muscles in full lumbar flexion. Narouei et al. [4], who analyzed surface EMG and kinematic data in people performing Nordic hamstring exercise (a dynamic lengthening hamstring exercise that requires trunk and hip muscle activation), confirmed the importance of synergistic muscles and trunk muscle coactivation in eccentric and concentric hamstring contractions. Our study demonstrated that an eight-week regimen combining active hamstring stretching with core stabilization exercises significantly alleviated LBP and low back discomfort associated with prolonged sitting in young adults. The experimental group exhibited statistically significant improvements in several key parameters as compared to the control group, including reductions in pain intensity VAS, disability (ODI), and low back discomfort (LBD), and enhancements in lower extremity range of motion in the SLR test. Similarly, Shamsi et al. [5] suggested that in LBP sufferers, both static stretching and strengthening of hamstring muscle in its lengthened position caused elongation and extensibility in the hamstring muscle and increased straight leg raising test score but did not change pelvic tilt angle. Czaprowski et al. [6], in their review on postural misalignments in the sagittal plane, indicate that not only the analysis of muscles with respect to their shortening and lengthening but also their hypoactivity and hyperactivity should be considered when planning therapeutic exercises, which also corresponds with our findings. Nonetheless, in contrast to those studies [5,6], we tested the intervention for person-centered outcomes, such as pain (VAS) and perceived disability (ODI). Marshall et al. [34], in their cross-sectional observation in twenty-one people with CLBP and fifteen controls, included VAS for pain and ODI for disability measurements. In contrast with our study, they doubt whether decreased hamstring extensibility should be targeted in rehabilitation programs in those patients. We present findings of a prospective, controlled trial, a study less prone to bias. In their randomized controlled trial, Park et al. [37] found, similarly to our study, that active exercises—in their study, those were motor control exercises—as well as stretching exercise (with more significant effects of the latter, with statistical differences at *p* < 0.05) led to improvements in LBP, as measured with VAS.

These findings underscore the potential of targeted exercise programs in managing NLBP among young adults who are prone to prolonged sitting. The improvements observed in the experimental group suggest that increasing hamstring flexibility and enhancing core stability can play a crucial role in reducing lumbar spine strain and discomfort during prolonged periods of sitting. This has significant implications for the development of preventive and therapeutic exercise protocols for NLBP management in comparable populations. A systematic review of prospective cohort studies from 2017 found an association between a restriction in lateral flexion and hamstring range of motion as well as limited lumbar lordosis with an increased risk of developing LBP in a total of 5459 heterogeneous, healthy participants [19], while a recent (2023) controlled trial claimed that, in a hundred people with CLBP, self-administered stretching exercises are as effective as motor control exercises [21]. The self-stretching exercise group in that trial performed six stretches in 40 min sessions, while the motor control exercise group performed trunk stabilizing exercises in 40 min sessions, and both groups performed weekly supervised sessions for 8 weeks [21]. We decided to introduce an easy, efficient program that can be carried out at home for young people (Generation Z) who have difficulties with long-lasting challenges. Home-based exercises are a promising approach to the management of LBP because they require fewer resources and less time from health institutions and health practitioners. A systematic review and meta-analysis by Quentin and colleagues [40] showed that exercise training to improve NLBP which takes place at home can lead to an improvement in pain and functional limitation. Moreover, home-based exercise training could be a cost-effective intervention in the treatment of NLBP.

The study’s results can be attributed to the specific physiological benefits of the intervention. Active hamstring stretching helps reduce muscle tension and increase flexibility, which can decrease the stress on the lumbar spine during movements involving hip flexion. Core stabilization exercises, on the other hand, strengthen the muscles supporting the spine, leading to better postural control and reduced compensatory mechanisms that often result in discomfort and pain.

The outcomes of this study align with previous research highlighting the benefits of flexibility and core strengthening exercises in the management of NLBP. Numerous studies have reported similar findings, reinforcing the effectiveness of these types of interventions in improving musculoskeletal health and reducing pain [5,20,21]. For instance, previous research has established that poor hamstring flexibility and weak core muscles contribute significantly to LBP, and targeted hamstring stretching exercise programs can mitigate these issues effectively.

Despite the positive outcomes, the study has several limitations. The relatively small sample size and the short duration of the intervention limit the generalizability of the results. Larger trials in homogenous populations, with long follow-ups, are warranted. Nonetheless, given the global burden of low back pain as a leading cause of years lived with disability (YLD), the generic rather than specific definition of low back pain (i.e., caution is needed regarding the distinction between specific and unspecific and acute and chronic LBP), as well as constraints regarding primary country-level data on low back pain on both prevalence and severity distributions, the external validity of a single trial will be limited to populations similar to the one studied, both in terms of intrinsic factors, such as BMI, age, and physical activity, and extrinsic ones, such as country or region [14,15,16,17,18]. The presented study focused exclusively on young adults, who may not be representative of other age groups or populations with different baseline physical activity levels and health conditions. Moreover, the study did not assess the long-term sustainability of the intervention’s benefits, which is critical for understanding its efficacy over time. Future studies should aim to include larger and more diverse populations to enhance the external validity and generalizability of the findings. Longitudinal research is also necessary to evaluate the long-term effects of such interventions. Additionally, examining the impact of similar exercise protocols on different age groups and individuals with varying physical activity levels would provide a more comprehensive understanding of the intervention’s overall effectiveness. The exploration of the integration of these exercises into daily routines and their potential use in preventive health strategies could also be valuable.

In future research, we plan to investigate various versions of the intervention, such as varied durations, frequency, and combinations with other therapeutic activities, to determine the most effective protocols for reducing NLBP and musculoskeletal discomfort. This corresponds with other research. Quentin et al., in a current (2021) meta-analysis, found considerable evidence that home-based exercise training improves pain intensity and functional limitation parameters in people with LBP but also found evidence gaps and primary study limitations regarding the duration and frequency of the sessions and programs [23]. A latest tertiary study addressing current clinical practice guidelines for LBP found that, as regards CLBP, guidelines from various countries and regions provide recommendations for exercise treatment but are significantly heterogeneous as regards exact recommendations [42], so studies testing specific exercise regimes and parameters are warranted. Two more issues, regarding, on the one hand, the methodology of exercise trials and internal validity [43], and on the other, population studies [14,15,16] and policy factors such as guideline development [42], as well as screening and preventive strategies and their limitations [44], should also be considered when planning and designing future studies.

Despite these limitations, our findings correspond with, and add to, evidence-based recommendations for managing and preventing low back pain [42]. This study aims to fill a specific research gap by examining the effectiveness of active hamstring stretching exercises combined with hip flexion mobilization in reducing NLBP and perceived musculoskeletal discomfort during prolonged sitting in young adults. Existing NLBP therapies usually concentrate on general flexibility and strengthening exercises without targeting specific muscle groups, such as the hamstrings, in addition to hip flexion procedures. This study fills a gap by examining a focused intervention that may provide a more effective strategy to addressing NLBP and discomfort in young adults, a cohort that is increasingly afflicted by sedentary lifestyles and prolonged sitting.

## 5. Conclusions

The study provides compelling evidence that an eight-week program of active hamstring stretching and core stabilization exercises can significantly reduce NLBP and musculoskeletal discomfort during prolonged sitting in young adults. These findings highlight the importance of targeted exercise interventions in managing and preventing NLBP, particularly among populations engaged in sedentary activities. Further research is warranted to confirm these findings and explore their applicability to broader populations and over extended periods.

## Figures and Tables

**Table 1:** Pre-intervention characteristics of participants.

Characteristics	E-Group	C-Group	*p*-Value
Demographics			
Female, n (%)	18 (69.2%)	16 (61.5%)	0.5599
Male, n (%)	8 (30.8%)	10 (38.5%)	
Body weight (kg)	69.2 (11.5)	171.5 (9.3)	0.9595
Body height (cm)	69.4 (16.3)	172.5 (10.3)	0.7144
Spinal curve angles			
Kyphotic angle VP-ITL [°] in a StP	40.8 (6.8)	42.9 (8.4)	0.3748
Lordotic angle ITL-DM [°] in a StP	33.7 (9.7)	38.7 (10.6)	0.0805
Kyphotic angle VP-ITL [°] in a SitP	41.5 (10.2)	39.3 (9.5)	0.3506
Lordotic angle ITL-DM [°] in a SitP	2.4 (5.3)	5.1 (4.4.)	0.0905
Baseline outcome measure characteristics			
SLR RL	68.9 (6.6)	70.8 (3.1)	0.6890
SLR LL	69.4 (5.8)	71.3 (1.9)	0.9926
VAS	5.8 (1.3)	5.3 (1.2)	0.0854
ODI	11.7 (6.7)	12.5 (3.6)	0.1850
LBD	6.0 (2.4)	5.1 (1.8)	0.1112

Data presented as mean (SD) unless otherwise stated (sex). E-group—Experimental group; C-group—Control group; StP—standing position; SitP—sitting position; SLR—straight leg raise, RL—right leg, LL—left leg; VAS—Visual Analogue Scale; ODI—Oswestry Disability Index; LBD—low back discomfort. Statistical significance was set at *p* < 0.05.

**Table 2:** The frequency of NLBP, the circumstances in which it occurred, and the knowledge of ergonomics among the participants.

		E-Group	C-Group
The frequency of NLBP			
Very rare NLBP (1–3/month)	n (%)	16 (61.5%)	12 (46.2%)
NLBP a few times a month (1–3/week)	n (%)	9 (34.6%)	12 (46.2%)
Frequent or constant NLBP (more than 3/week)	n (%)	1 (3.9%)	2 (7.7%)
Circumstances in which NLBP occurs or increases *			
Sitting	n (%)	18 (69.2%)	18 (69.2%)
Standing	n (%)	16 (61.5%)	12 (46.2%)
Lying	n (%)	4 (15.4%)	8 (30.8%)
Lifting heavy objects	n (%)	8 (30.8%)	8 (30.8%)
Performing household chores (cleaning, cooking, getting dressed)	n (%)	4 (15.4%)	12 (46.2%)
Physical effort	n (%)	6 (23.1%)	4 (15.4%)
The knowledge of how to sit properly			
I don’t know the rules	n (%)	2 (7.7%)	6 (23.1%)
Yes, I know the rules, but I can’t apply them in practice	n (%)	12 (46.2%)	8 (30.8%)
Yes, I know the rules and I can apply them in practice	n (%)	12 (46.2%)	12 (46.2%)

* The numbers do not add up to 100% since the respondents were allowed to choose more than one answer; E-group—Experimental group; C-group—Control group.

**Table 3:** Results for the outcomes pre- and post-intervention observed in the E-group and C-group.

		E-Group	p-Value	C-Group	p-Value
VAS	Pre	5.8 (1.3)	**0.0458**	5.3 (1.2)	0.1128
	Post	5.3 (1.6)		5.7 (1.1)	
ODI	Pre	11.7 (6.7)	**0.0479**	12.5 (3.6)	0.1401
	Post	9.5 (4.9)		14.0 (6.1)	
LBD	Pre	6.0 (2.4)	**0.0137**	5.1 (1.8)	0.3078
	Post	4.6 (2.7)		4.6 (1.8)	
SLR RL	Pre	68.9 (5.3)	**0.0000**	68.7 (2.2)	0.5098
	Post	74.2 (4.9)		68.9 (2.5)	
SLR LL	Pre	67.3 (4.3)	**0.0000**	69.7 (0.5)	0.3280
	Post	74.1 (4.0)		70.0 (1.5)	

Data presented as mean (SD). SD—standard deviation; E-group—Experimental group; C-group—Control group; VAS—Visual Analogue Scale; ODI—Oswestry Disability Index; LBD—low back discomfort; SLR—straight leg raise, RL—right leg, LL—left leg. Statistical significance was set at *p* < 0.05, statistically significant differences are presented in bold.

**Table 4:** Post-intervention inter-group differences.

	E-Group	C-Group	*p*-Value
VAS, Δ (95% CI)	0.5 (−0.1 to 1.1)	−0.4 (−0.8 to 0.0)	**0.0367**
ODI, Δ (95% CI)	2.2 (−0.2 to 4.5)	−1.5 (−3.7 to 0.6)	0.0670
LBD, Δ (95% CI)	1.0 (0.3 to 1.7)	0.2 to (−0.2 to 0.7)	**0.0063**
SLR RL, Δ (95% CI)	7.5 (6.5 to8.5)	0.2 (−0.5 to 0.9)	**0.0000**
SLR LL, Δ (95% CI)	6.8 (5.6 to 7.9)	0.3 (−0.3 to 1.0)	**0.0000**
GPE, mean (SD)	2.8 (1.0)	3.4 (1.0)	**0.0346**

Data presented as delta Δ (CI) and mean (SD). E-group—Experimental group; C-group—Control group; CI—confidence interval; VAS—Visual Analogue Scale; ODI—Oswestry Disability Index; LBD—low back discomfort; SLR—straight leg raise, RL—right leg, LL—left leg; GPE—Global Perceived Effect. Statistical significance was set at *p* < 0.05, statistically significant differences are presented in bold. Positive delta values indicate a decrease in the values of the VAS, ODI, and LBD parameters and an increase in the values of the SLR test.

## Data Availability

Data are contained within the article.
